# 3D genome contributes to MHC-II neoantigen prediction

**DOI:** 10.1186/s12864-024-10687-3

**Published:** 2024-09-26

**Authors:** Mofan Feng, Liangjie Liu, Kai Su, Xianbin Su, Luming Meng, Zehua Guo, Dan Cao, Jiayi Wang, Guang He, Yi Shi

**Affiliations:** 1 Bio-X Institutes, Key Laboratory for the Genetics of Developmental and Neuropsychiatric Disorders, Shanghai Jiao Tong University, 1954 Huashan Road, Shanghai, 200030 China; 2 Shanghai Key Laboratory of Psychotic Disorders, and Brain Science and Technology Research Center, Shanghai Jiao Tong University, 1954 Huashan Road, Shanghai, 200030 China; 3 Key Laboratory of Systems Biomedicine, Shanghai Center for Systems Biomedicine, Ministry of Education, Shanghai Jiaotong University, Shanghai, 200240 China; 4 College of Biophotonics, South China Normal University, Guangzhou, 510631 China; 5 Department of Instrument Science and Engineering, School of Electronic Information and Electrical Engineering, Shanghai Jiao Tong University, Shanghai, 200240 China; 6 Department of Obstetrics and Gynecology, International Peace Maternity and Child Health Hospital, Shanghai Jiaotong University School of Medicine, Shanghai, 200030 China; 7 Shanghai Institute of Thoracic Oncology, Shanghai Chest Hospital, Shanghai Jiaotong University, Shanghai, 200030 China; 8 eHealth Program of Shanghai Anti-Doping Laboratory, Shanghai University of Sport, Shanghai, 200438 China

**Keywords:** HLA-II, pMHC-II, CD4, Neoantigen, 3D genome, HiC, Immunotherapy, Epitope

## Abstract

Reliable and ultra-fast DNA and RNA sequencing have been achieved with the emergence of high-throughput sequencing technology. When combining the results of DNA and RNA sequencing for tumor cells of cancer patients, neoantigens that potentially stimulate the immune response of either CD4^+^ or CD8^+^ T cells can be identified. However, due to the abundance of somatic mutations and the high polymorphic nature of human leukocyte antigen (HLA) it is challenging to accurately predict the neoantigens. Moreover, comparing to HLA-I presented peptides, the HLA-II presented peptides are more variable in length, making the prediction of HLA-II loaded neoantigens even harder. A number of computational approaches have been proposed to address this issue but none of them considers the DNA origin of the neoantigens from the perspective of 3D genome. Here we investigate the DNA origins of the immune-positive and non-negative HLA-II neoantigens in the context of 3D genome and discovered that the chromatin 3D architecture plays an important role in more effective HLA-II neoantigen prediction. We believe that the 3D genome information will help to increase the precision of HLA-II neoantigen discovery and eventually benefit precision and personalized medicine in cancer immunotherapy.

## Introduction

Immunotherapies through boosting the endogenous T cell’s ability to destroying cancer cells have demonstrated effective in a variety of human malignancies [1]. The mutations of cancers can encode the seeds of their own destruction, in the form of T cell recognizable immunogenic peptides, also known as neoantigenic epitopes. There are two major origins of cancer rejection epitopes: the first origin of such antigens is formed by non-mutated proteins to which T cell tolerance is incomplete due to their restricted tissue expression pattern; the second origin is formed by peptides that are novel in normal human genome, known as neoantigens [1]. With the advance of sequencing technology, it has been revealed that during cancer development, a large number of somatic mutations can be generated. Most of these mutations are caused by genomic instability within the tumor cells and are invidious passenger mutations with unobvious growth advantage; a limited number of cancer mutations however, are driver mutations which interfere with normal cell regulation and can contribute to cancer growth and resistance to targeted therapies [2]. Both passenger mutations and driver mutations can be nonsynonymous that alter protein amino acid sequence coding, leading tumor to express abnormal proteins that cannot be found in normal cells. During cell metabolize, the proteins possessing abnormal sequences are trimmed into short peptides and are presented on the cell surface by the major histocompatibility complex (MHC, or HLA in humans) which have a chance to be recognizable by T cell as foreign antigens [2–4].

The cancer-killing ability of CD8^+^ T cells (killer T cell) can be stimulated when the T cell receptors (TCRs) recognize cancerous peptide epitopes that are displayed on major histocompatibility complex-I (MHC-I, HLA-I in human) on the surface of the tumor cells. CD4^+^ T cells on the other hand, identify peptides bound to MHC-II (HLA-II in human) molecules displayed on the surface of antigen-presenting cells (APCs). In recent years, CD4^+^ T cell (helper T cell) is drawing more attention in cancer immunotherapy area, as studies have shown that in infections or cancer, when non-self peptides or tumor-associated antigens are generated, interactions between the HLA-II–peptide complex on APCs and the TCR on CD4^+^ T cells, are key to initiate and sustain immune responses [5–7]. Comparing to HLA-I presented neoantigen discovery, due to the high polymorphic nature of HLA-II and that the HLA-II presented peptides are more variable in length, it is more challenging to efficiently predict the HLA-II loaded neoantigens [8–10].

According to the above principles, if candidate neoantigens are identified via sequencing experiment, one can validate the efficacy of the synthesized epitope peptides in vivo (cancer cell-line or animal model) before clinical practice [1, 2] and indeed, cancers bearing sporadic dominant mutation can often be effectively treated by targeting the driver mutation [2, 11]. When the somatic mutations are abundant however, which is a more general scenario, it is challenging to efficiently prioritize the identified neoantigen candidates according to their ability to activate the T cell’s immuno-response [12]. Although HLA peptidomics development in recent years [13–15] allow fast and reliable measurements of thousands of HLA ligands per sample, which improve HLA-I epitope predictions to a large extend [16–20], similar improvements are not hold for HLA-II, and previous studies based on high-throughput peptidomics have been restricted to a few HLA-II alleles [14, 21] or failed to demonstrate improvements in epitope predictions at all [22], leaving the space of computational HLA-II neoantigen prediction still wide open.

Over the past two decades, numerous neoantigen prediction approaches have been proposed [8, 9, 19, 23, 24] which can be partitioned into two major categories: the protein 3D structure-based approaches which consider the 3D conformations of pMHC and TCR, and the sequence-based approaches which consider the amino acid sequence of the target peptides. For the 3D structure-based approaches, if high quality pMHC 3D structures are available, molecular dynamic (MD) methods can be applied to investigate the contact affinity of pMHC-TCR complex [25–27], otherwise the modelling or simulation by protein docking and threading has to be employed due to the lack of high quality pMHC 3D conformation. Most other approaches adopt the sequence-based methods as there are much larger training datasets [28, 29] and the sequence-based approaches are usually more efficient to set up [12, 30].

Early sequence-based methods such as BIMAS [31] and SYFPEITHI [32] adopted the position-specific scoring matrices (PSSMs) which are defined from experimentally confirmed peptide binders of a particular MHC allele [12]. More advanced machine-learning based techniques were then developed to capture the nonlinear nature of the pMHC-TCR interaction which demonstrated better performance than the PSSM-based methods. Consensus approaches such as CONSENSUS [33] and NetMHCcons [34] that combine multiple methods were also developed to achieve more robust predictions, trading off additional computational power in determining the weighting among results generated by different methods. When considering peptide binding, most methods did not consider the HLA allele variety, therefore, pan-specific methods, such as NetMHCpan [19, 24], were developed which allow the HLA type independent prioritization. As one of the most widely adopted methods in the area, NetMHCpan first train a neural network based on multiple public datasets, then the affinity of a given peptide-MHC considering the polymorphic HLA types HLA-A, HLA-B or HLA-C is computed according to the trained neural network. NetMHCpan [19] and NetMHCIIpan [35] perform remarkably, even compared to allele-specific approaches [12, 36]. However, although several assessments and criteria were proposed in the past aiming at a more fair and effective comparison [36–38], there are no recent independent benchmark studies that can be used to recommend specific tools up until now [3].

In our previous study, we discovered that the DNA loci of MHC-I neoantigens obey certain distribution in genome 3D space [3] and by incorporating this important information, we developed a group feature selection based deep neural network model (DNN-GFS) that was able to predict MHC-I neoantigen in a much higher accuracy than the existing widely adopted methods [4]. To the best of our knowledge however, none of the present MHC-II epitope prediction methods consider the corresponding DNA loci of the neoantigens in the perspective of 3D genome, which carries important additional information compared to the amino acid sequence alone [39]. In this work, we incorporated the DNA origin of the immune-positive and non-negative MHC-II neoantigens in the context of the 3D genome and demonstrate its contribution to the MHC-II neoantigen prediction.

## Methods

### Immunogenicity data collection and curation

For MHC-II neoantigen training data, peptide sequences and the corresponding immune response information were collected from the IEDB database under the T-Cell Assay category [29] in May 2021. After collecting 399,318 peptide records in the primary dataset, we performed filtering by targeting Homo Sapiens species and MHC-II subtypes, and restrained the peptide length from 11 to 30, followed by identical records (i.e., same peptide sequence and HLA subtype) merging. The dataset was further cleaned up by applying two procedures, *checkIllegalPeptides* and *modifyHLAType*, which checks for amino-acid alphabet legitimacy and standardizes HLA allele names, respectively. For peptides of unknown MHC subtype, DRB1*01:01 was set as default MHC allele. For peptides of known MHC subtypes, we sorted them into different datasets for separate training and evaluation. Identical peptides with multiple immune experiments are defined as immuno-positive or immuno-negative if the positive rate > 70% or the positive rate < 30%, respectively. In the end, we obtained 3,633 peptides, with 2,197 immuno-negative and 1,436 immuno-positive. As for the sub data set which contains detailed MHC-II subtype information, there were in total 703 peptides, of which 411 are immuno-positive ones and 292 are immuno-negative ones.

### Mapping peptides to human genome

We developed a pipeline to map the peptides sequence to reference human genome hg19; the pipeline query the NCBI local BLAST [40] and map the gene names to chromosomes and start-end positions. To set up local BLAST, we restricted the search to H.sapiens and set the E-value to 0.01 to find matches. After obtaining the accessions, we used the BIOMART [41] to convert the gene name to ENSEMBL ID, then we used the DAVID [42] to obtain the gene names composed with gene symbols and the chromosome positions were then obtained.

### Chromatin 3D modeling

We used the contact frequency Hi-C data of the hESC and IMR90 cell lines generated by Bin Ren’s lab as the chromatin 3D conformation data source [43]. The contact frequencies and the subsequent chromatin 3D modeling are based on these population cell based Hi-C data. We developed a whole-genome 3D modeling algorithm for the human genome using molecular dynamics (MD) based approach with resolution of 500 kb (bin size) for hESC and IMR90 Hi-C data. Each bin was coarse-grained by the algorithm as one bead and intact genome was modeled as 23 polymer chains represented by bead-on-the-string structures [4]. Two factors would affect the spatial position of each bead: the chromatin connectivity that constrains sequentially neighbor beads in close spatial proximity and the chromatin activity that ensures active regions are more likely to be located close to the center of cell nucleus [4]. We estimated the chromatin activity as compartment degree that can be directly calculated from Hi-C matrix with algorithm described in previous work [44]. All the beads were assigned distances to the nuclear center and the conformation of chromatin was optimized from random initial structures using MD approach. The bias potential was applied to satisfy the distance constraints.

### MHC-II neoantigen prediction method

We adopted the NetMHCIIpan method with Binding Affinity (BA) and Mass-Spectrometry Eluted Ligands (EL) training means respectively, to predict the curated peptides’ immunogenicity as baseline predictions. The predicted results were then treated as input feature along with the 3D genome coordinates and radius position values of hESC and IMR90. The 9 input features (1 of BA or EL, 6 of < x, y, z > coordinates from hESC and IMR90 3D models, and 2 of radius positions from hESC and IMR90 3D models) were then taken together to train KNN (K-Nearest Neighbor) with K = 8 after parameter tuning, SVM (Support Vector Machine) with default parameter of Gaussian kernel, and LR (Logistic Regression), under 5-fold and leave-one-out (LOO) cross validation schemes, to validate its contribution to the baseline prediction.

## Results

**Fig. 1:**
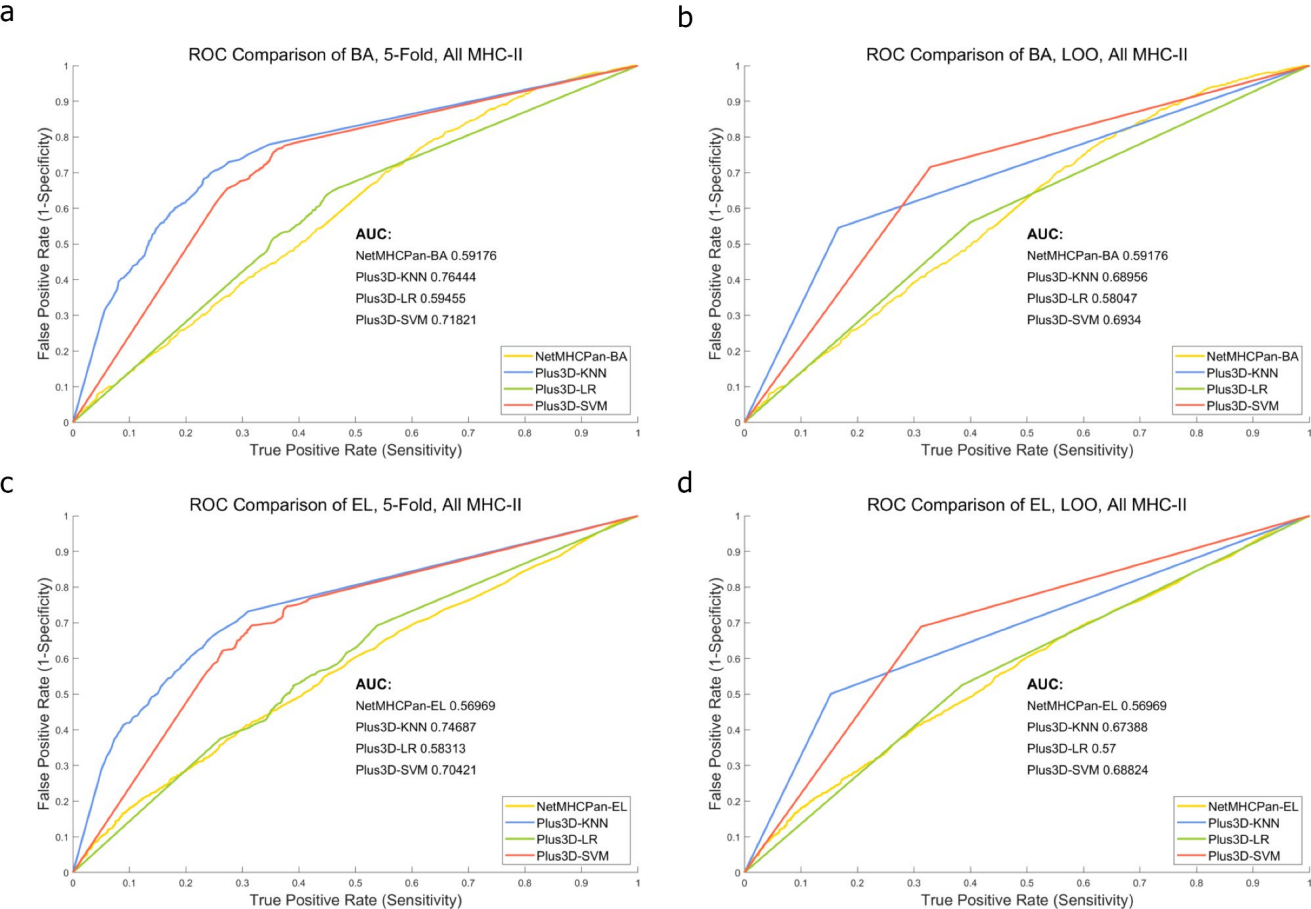
ROC curve comparison of different prediction methods applied on the 3,633-peptide dataset. **a** and **b**: NetMHCPan-BA results are adopted as baseline predictions and are compared with Plus3D-KNN, Plus3D-LR and Plus3D-SVM predictions, under 5-fold and Leave-one-out cross validations respectively. **c** and **d**: NetMHCPan-EL results are adopted as baseline predictions and are compared with Plus3D-KNN, Plus3D-LR and Plus3D-SVM predictions, under 5-fold and Leave-one-out cross validations respectively

**Fig. 2:**
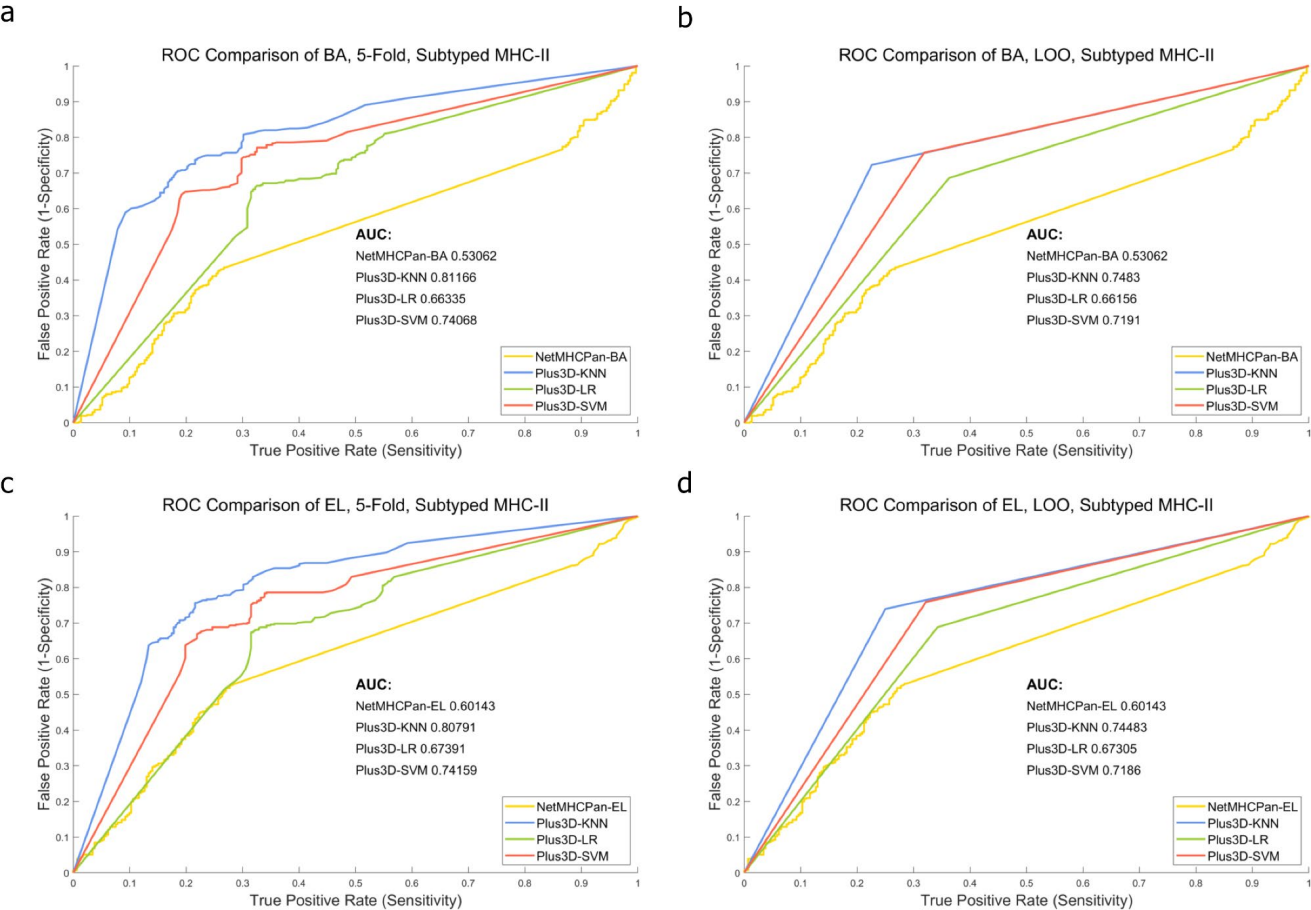
ROC curve comparison of different prediction methods applied on the 703-peptide dataset. **a** and **b**: NetMHCPan-BA results are adopted as baseline predictions and are compared with Plus3D-KNN, Plus3D-LR and Plus3D-SVM predictions, under 5-fold and Leave-one-out cross validations respectively. **c** and **d**: NetMHCPan-EL results are adopted as baseline predictions and are compared with Plus3D-KNN, Plus3D-LR and Plus3D-SVM predictions, under 5-fold and Leave-one-out cross validations respectively

**Fig. 3:**
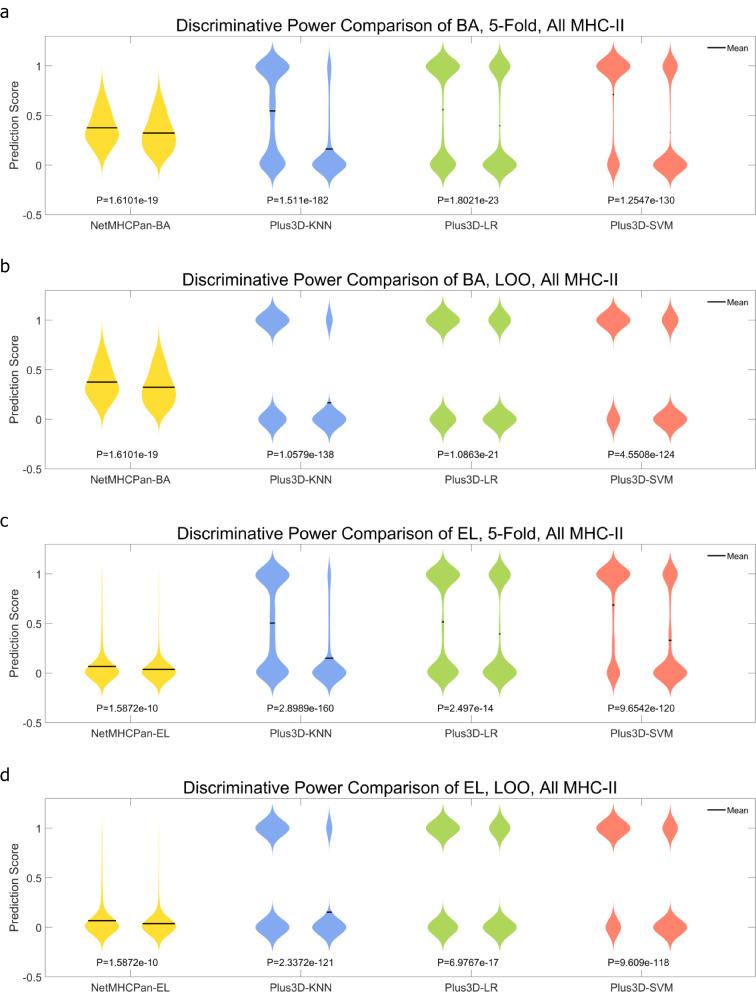
Positive and negative sample prediction score distribution comparison of different prediction methods applied on the 3,633-peptide dataset. **a** and **b**: NetMHCPan-BA results as baseline predictions are compared with Plus3D-KNN, Plus3D-LR and Plus3D-SVM predictions, under 5-fold and LOO cross validations respectively. **c** and **d**: NetMHCPan-EL results as baseline predictions are compared with Plus3D-KNN, Plus3D-LR and Plus3D-SVM predictions, under 5-fold and LOO cross validations respectively

**Fig. 4:**
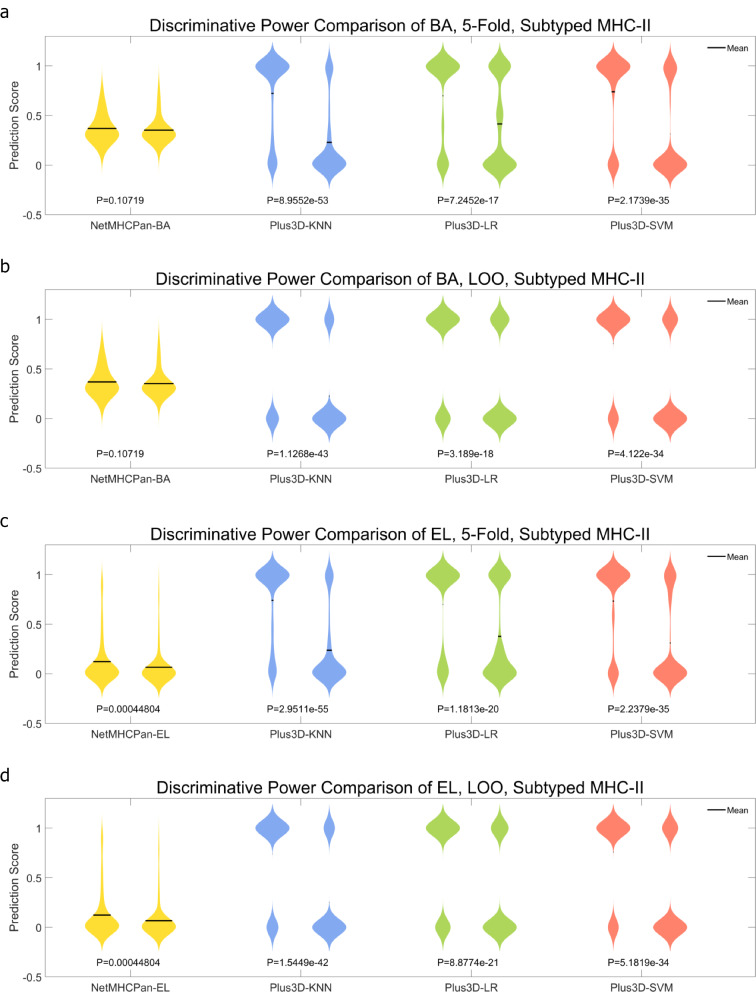
Positive and negative sample prediction score distribution comparison of different prediction methods applied on the 703-peptide dataset. **a** and **b**: NetMHCPan-BA results as baseline predictions are compared with Plus3D-KNN, Plus3D-LR and Plus3D-SVM predictions, under 5-fold and LOO cross validations respectively. **c** and **d**: NetMHCPan-EL results as baseline predictions are compared with Plus3D-KNN, Plus3D-LR and Plus3D-SVM predictions, under 5-fold and LOO cross validations respectively

After curation and generation of the two datasets, i.e., the 3,633-peptide one which contains both known and unknown MHC-II subtype p-MHCs, and the 703-peptide one which contains only known MHC-II subtype p-MHCs. We first run NetMHCPan-BA and NetMHCPan-EL to generate the NetMHCPan results as baseline predictions. We then incorporated 3D genome features, i.e., the < x, y, z > coordinates and the radius position of both hESC and IMR90 Hi-C cell lines, and trained KNN, SVM, and LR models to obtain final predictions under either 5-fold (100 repeats) or LOO cross validations. Figures 1 and 2 demonstrate the ROC curve comparison in 3,633-peptide dataset and 703-peptide dataset, respectively. In either Figure, NetMHCPan-BA or NetMHCPan-EL are the baseline predictions and Plus3D-KNN, Plus3D-LR and Plus3D-SVM are predictions after incorporating 3D genome features under cross validations. Figures 3 and 4 are the prediction score compassion of different methods under known positive and negative immunogenicity category, to demonstrate the discriminative power of different methods. The figures clearly demonstrate that after incorporating 3D genome information, the prediction accuracies are significantly boosted no matter what prediction method are used, indicating that 3D genome information can contribute more precise p-MHC-II neoantigen prediction to a large extend. Tables 1 and 2 demonstrated detailed prediction statistics at the cutoffs that reach the best F-measure score for each situation.

**Table 1:** Statistics of different prediction methods applied on the 3633-peptide dataset

Setting	Method	Precision	Recall	F1-measure	AUPR	AUC
**BA**,** 5Fold**	NetMHCPan-BA	0.4498	**0.7618**	0.5857	0.461	0.5918
	Plus3D-KNN	**0.6889**	0.5905	**0.6752**	**0.7167**	**0.7644**
	Plus3D-LR	0.4811	0.6372	0.5666	0.5876	0.5946
	Plus3D-SVM	0.6101	0.656	0.6605	0.7068	0.7182
**BA**,** LOO**	NetMHCPan-BA	0.4498	**0.7618**	0.5857	0.461	0.5918
	Plus3D-KNN	**0.6821**	0.5453	0.606	0.7035	0.6896
	Plus3D-LR	0.4783	0.5606	0.5666	0.6063	0.5805
	Plus3D-SVM	0.5871	0.7159	**0.6451**	**0.7076**	**0.6934**
**EL**,** 5Fold**	NetMHCPan-EL	0.3953	**1.0**	0.5666	0.4808	0.5697
	Plus3D-KNN	**0.6831**	0.5313	**0.6633**	**0.7046**	**0.7469**
	Plus3D-LR	0.4568	0.6915	0.5666	0.5617	0.5831
	Plus3D-SVM	0.6035	0.6233	0.6393	0.6873	0.7042
**EL**,** LOO**	NetMHCPan-EL	0.3953	**1.0**	0.5666	0.4808	0.5697
	Plus3D-KNN	**0.6815**	0.5007	0.5773	0.6898	0.6739
	Plus3D-LR	0.4712	0.5251	0.5666	0.592	0.57
	Plus3D-SVM	0.5904	0.6887	**0.6358**	**0.7011**	**0.6882**

**Table 2:** Statistics of different prediction methods applied on the 703-peptide dataset

Setting	Method	Precision	Recall	F1-measure	AUPR	AUC
**BA**,** 5Fold**	NetMHCPan-BA	0.5855	**1.0**	0.7385	0.6222	0.5306
	Plus3D-KNN	**0.8446**	0.7007	**0.799**	**0.8664**	**0.8117**
	Plus3D-LR	0.7418	0.6642	0.7379	0.7921	0.6634
	Plus3D-SVM	0.821	0.6472	0.7704	0.8365	0.7407
**BA**,** LOO**	NetMHCPan-BA	0.5855	**1.0**	0.7385	0.6222	0.5306
	Plus3D-KNN	**0.8182**	0.7226	**0.7674**	**0.8515**	**0.7483**
	Plus3D-LR	0.7268	0.6861	0.7379	0.7982	0.6616
	Plus3D-SVM	0.7698	0.7567	0.7632	0.8344	0.7191
**EL**,** 5Fold**	NetMHCPan-EL	0.5846	**1.0**	0.7379	0.6812	0.6014
	Plus3D-KNN	**0.8293**	0.7567	**0.8102**	**0.8702**	**0.8079**
	Plus3D-LR	0.7487	0.6813	0.7429	0.7891	0.6739
	Plus3D-SVM	0.804	0.6788	0.7746	0.8361	0.7416
**EL**, **LOO**	NetMHCPan-EL	0.5846	**1.0**	0.7379	0.6812	0.6014
	Plus3D-KNN	**0.8064**	0.7397	**0.7716**	**0.8491**	**0.7448**
	Plus3D-LR	0.7389	0.6886	0.7379	0.8048	0.673
	Plus3D-SVM	0.7685	0.7591	0.7638	0.8342	0.7186

## Discussion

The neoantigen therapy is a rising and promising strategy in cancer immunotherapy area, as it can be absolutely personalized and catch up with cancer evolution by updating neoantigen panel. It is computationally challenging however, to efficiently predict neoantigen candidates according to their ability of activating the T cell immuno-response, especially when the somatic mutations are abundant. Dozens of neoantigen prioritization or prediction approaches have been proposed to address this issue for either MHC-I or MHC-II presented epitopes, corresponding to CD8^+^ and CD4^+^ T cells respectively, but none of the existing approaches considers the DNA origin of the neoantigens from the perspective of 3D genome. In this work, we demonstrated that similar to our previous discovery for MHC-I and CD8^+^ T cell neoantigen, the 3D genome information can contribute to much more accurate MHC-II neoantigen prediction. The underlining mechanism why 3D genome is closely linked to neoantigen immunogenicity is yet to be revealed, but here we conjecture that it is the evolution of chromatin 3D conformation that positioned protein-coding DNA segments of different immunogenicity-activating power in specific locations in the 3D genome within the nucleus.

One perspective that worth further investigation is how the 3D genome contributes to neoantigen immunogenicity prediction by offering information into the evolutionary dynamics of genes and their regulatory elements within the nucleus. Better understanding of the 3D genome architecture allows researchers to identify how genetic elements, such as enhancers and promoters, have evolved to regulate the expression of genes. Evolution shapes these regulatory elements over time, enabling organisms to adapt and respond to different environmental and physiological conditions. When considering neoantigen immunogenicity prediction, the 3D genome can help identify the evolutionary changes that have occurred in regulatory elements controlling the expression of genes encoding tumor-specific neoantigens. Moreover, 3D genome sub-architectures such as A/B Compartment, TAD, Loop can also be further investigated along with neoantigen immunogenicity, for both MHC-I and MHC-II neoantigens, and better prediction models can be achieved by incorporating such information.

We believe that by incorporating the 3D genome information better, e.g., combining more advanced machine learning [45–47] and feature selection technologies [48–50], more precise neoantigen prioritization and discovery can be achieved and will eventually benefit precision medicine in cancer immunotherapy.

## Data Availability

Data and materials will be updated on https://yishi.sjtu.edu.cn/deepAntigen/.

## Declarations

### About this supplement

This article has been published as part of BMC Genomics, Volume 25 Supplement S2, 2024: Selected articles from the 18th International Symposium on Bioinformatics Research and Applications (ISBRA-22). The full contents of the supplement are available at https://bmcgenomics.biomedcentral.com/articles/supplements/volume-25-supplement-2.

## Abbreviations

MHC: Major Histocompatibility Complex
HLA: Human leukocyte antigens
TCR: T-Cell Receptor
IEDB: The Immune Epitope Database
BLAST: Basic Local Alignment Search Tool
MD: Molecular Dynamic
KNN: K-Nearest Neighbor
SVM: Support Vector Machine
LR: Logistic Regression
LOO-CV: Leave One Out Cross Validation
